# Differences in Patient Age Distribution between Influenza A Subtypes

**DOI:** 10.1371/journal.pone.0006832

**Published:** 2009-08-31

**Authors:** Hossein Khiabanian, Gregory M. Farrell, Kirsten St. George, Raul Rabadan

**Affiliations:** 1 Department of Biomedical Informatics and Center for Computational Biology and Bioinformatics, Columbia University College of Physicians and Surgeons, New York, New York, United States of America; 2 Laboratory of Viral Diseases, Wadsworth Center, New York State Department of Health, Albany, New York, United States of America; INSERM, France

## Abstract

Since the spring of 1977, two subtypes of influenza A virus (H3N2 and H1N1) have been seasonally infecting the human population. In this work we study the distribution of patient ages within the populations that exhibit the symptomatic disease caused by each of the different subtypes of seasonal influenza viruses. When the publicly available extensive information is pooled across multiple geographical locations and seasons, striking differences emerge between these subtypes. We report that the symptomatic flu due to H1N1 is distributed mainly in a younger population relative to H3N2. (The median age of the H3N2 patients is 23 years while H1N1 patients are 9 years old.) These distinct characteristic spectra of age groups, possibly carried over from previous pandemics, are consistent with previous reports from various regional population studies and also findings on the evolutionary dynamics of each subtype. Moreover, they are relevant to age-related risk assessments, modeling of epidemiological networks for specific age groups, and age-specific vaccine design. Recently, a novel H1N1 virus has spread around the world. Preliminary reports suggest that this new strain causes symptomatic disease in the younger population in a similar fashion to the seasonal H1N1 strains.

## Introduction

The year 1918 was marked by the “Spanish flu” H1N1 pandemic that killed more than 50 million people worldwide [1]. The H1N1 virus disappeared from the human population nearly forty years later, when in 1957 the H2N2 pandemic propagated around the globe. Except for a few singular reports, such as the Fort Dix swine flu case in 1976 [2], H1N1 was not isolated from humans for another 20 years. In May of 1977, the human H1N1 strain reappeared in Northern China [3]. At the time, the main concern was for the younger population born after 1957, which had never been exposed to this subtype of the virus. As expected, the spreading epidemic was almost entirely restricted to the sub-adult population [4].

Due to improvements in public health conditions, the life expectancy in Europe and North America has increased. In contrast however, most probably because of the growing size of the older population, the US influenza death toll surged fourfold from 1976 to 1999. Older people, along with young children, are particularly vulnerable to severe outcomes and secondary infections [5], [6].

Over the last few years, the information available in various public influenza databases has expanded dramatically. This information, appended to the sequences of viral isolates, includes the age and sex of the patient, and the date and geographical location of specimen collection. Thus, the probing of databases to attempt to uncover demographic trends has become feasible. Furthermore, the statistical significance of the conclusions that are drawn is reinforced when the same trends are observed across independent datasets, and most particularly when datasets are merged in various combinations.

An important limitation for the collection of a truly random set of influenza isolates is the fact that not all persons infected by influenza virus clearly show the symptoms of influenza-like illness (ILI). Any strain of the virus can infect an individual without causing acute symptomatic disease. Thus, the content of the datasets is generally restricted to isolates from persons who were exhibiting at least fairly severe symptomatic disease, who are henceforth referred to as “patients” of a given subtype.

Past family and community-based studies, some testing the serum specimens for antibodies to the prevalent influenza viruses and some based on a broader interpretation of ILI symptoms, have demonstrated patterns in age-specific occurrence of illness caused by the different types and subtypes of influenza virus [7]–[11]. In particular, the Houston Family Study, which was conducted from 1977 through 1989, reported a significantly different age distributions of patients with H1N1 and H3N2 infections, where more than 50% of H1N1 infections were detected among the 10–34 years old patients and persons born before 1951 had a decreased risk of developing a virus-positive, medically attended illness. Glezen et al. [7] attribute this striking difference to the development of lasting immunity against H1N1 viruses due to exposure to previous epidemics.

Since March 2009 a new H1N1 strain of influenza A virus of swine origin has been infecting humans [12]. Most of the patients that show symptomatic disease from infection by this new strain are also young. In particular, Kelly et al. [13] report a similar median age of infection of 20±3 years for both the new and the seasonal strains,

In this work, we extended the analyses of age-specific occurrence of illness to geographically diverse populations within several recent influenza seasons. We analyzed samples from seasonal influenza in New York State collected by the Wadsworth Center, New York State Department of Health, and also datasets entailing temporally and geographically diverse information deposited in the Influenza Virus Resource of the National Center for Biotechnology Information (NCBI). [14] The vast majority of the data, deposited by the Influenza Genome Sequencing Project of the National Institute of Allergy and Infectious Diseases (NIAID), are from sequence strains that were not pre-selected for particular characteristics, such as subtype. [15] Therefore, the concern regarding unknown biases in sequence datasets can be mitigated by noting that although the samples were not methodically collected by a random protocol within the population, the number of incidents for both subtypes is sufficiently large that the bias in the study is reduced. In other words, because the sequences were not collected with a prior knowledge of the subtype of the infecting virus, the statistical significance of the conclusions solely depends on the quantities of the isolates for each subtype, and their comparability in number. Obviously, the larger the database, the higher the accuracy of probability estimates.

In this paper, we report a significant dissimilarity, relating to the patient age distribution of infection, between the two circulating influenza A subtypes and provide a more robust statistical measure by combining several datasets and comparing the age distributions of the influenza A subtypes. Our results are consistent with previous reports from various population studies and also findings on the evolutionary dynamics of each subtype.

## Methods

We first studied a dataset compiled by the Laboratory of Viral Diseases at the Wadsworth Center, New York State Department of Health (NYSDOH). This dataset, which includes the infecting virus subtype as well as the age and sex of the patient, spans the influenza A-positive specimens received during the 2006–2007 and 2007–2008 influenza seasons. There are a total of 77 H1N1 and 139 H3N2 isolates in this dataset, the majority of which are from sentinel physician submissions. The sentinel program, funded by the Centers for Disease Control and Prevention, has representative physicians enlisted throughout all regions of New York State, in various types of clinical practice, who submit specimens for testing from patients exhibiting influenza-like-illness throughout the season.

For a more extensive dataset, we acquired a set of sequences for the hemagglutinin (HA) segment from the H1N1 and H3N2 subtype isolates in the United States from the NCBI public database. We chose the HA segment solely because of the great number of available sequences. To try to ensure a high accuracy in the sequences, we selected ones from large-scale genome-sequencing projects, and we stipulated that the age and sex of the patient be available for each isolate. This U.S. dataset contains 512 H1N1 and 1168 H3N2 sequences collected as early as 1995, although the majority are from 2006–2008. Almost 700 of the submissions in this dataset from New York State were carefully selected for temporal and geographical diversity across each of the seasons from which they were submitted. Additionally, the majority of these samples originated from sentinel physician specimens submitted to the Wadsworth Center, as described above. By applying similar criteria for inclusion, we also acquired a set of HA sequences from Oceania (mostly from New Zealand) comprising 179 H1N1 and 586 H3N2 sequences collected from 2000 to 2007 (see Table 1).

**Table 1:** The studied datasets from New York State and the NCBI.

DataSet	Season	H1N1			H3N2			P(MW)*	P(KS)*
		Median^a^	Oldest^b^	Count^c^	Median^a^	Oldest^b^	Count^c^		
New York State	2006–2008	21	1947	77	24	1914	139	3.91E-05	4.81E-04
NCBI: United States	2007–2008	21	1948	136	25	1930	478	6.64E-03	5.18E-05
NCBI: United States	2006–2007	7	1940	299	13	1923	77	2.15E-06	1.70E-06
NCBI: United States	1995–2008	9	1928	512	26	1911	1168	3.56E-48	2.15E-50
NCBI: Oceania	2000–2007	20	1923	179	23	1907	586	4.19E-01	4.03E-05
NCBI: All Data	2000–2007	9	1923	583	23	1907	1014	3.77E-18	1.57E-25

a: the median age.

b: the birth year of the oldest person.

c: number of counts.

* Probabilities computed for Mann-Whitney (P(MN)) and Kolmogorov-Smirnov (P(KS)) tests.

As the information in both NYSDOH and NCBI datasets was de-identified, no approval from an ethics committee was necessary. Also, because the NCBI dataset is public and the NYSDOH dataset only provided the aggregated information, no patient consent was required.

The number of patients showing symptomatic disease in each of the age groups is a function of the particular characteristics of the analyzed subpopulation. For the purpose of assessing the age-trend differences between the groups contracting influenza caused by either the H1N1 or H3N2 subtype virus, we employed empirical cumulative distribution functions in relation to age. To assess the statistical significance of the trends, we chose the nonparametric Mann-Whitney and Kolmogorov-Smirnov tests; these respectively compare the two cumulative distributions via their ranking difference and their maximum difference.

## Results

**Figure 1:**
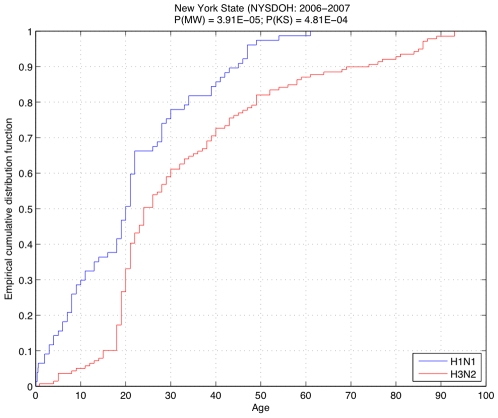
Empirical cumulative distribution of ages for patietns with H1N1 (blue) and H3N2 (red) in New York State during the 2006–2007 and 2007–2008 influenza seasons. The significantly low probabilities computed via Mann-Whitney (P(MN)) and Kolmogorov-Smirnov (P(KS)) tests indicate a remarkable dissimilarity between the distributions.

We first examined age trends in the NYSDOH dataset. When NYSDOH data from the 2006–2007 and 2007–2008 influenza seasons were combined, we found that 47% of the detected H1N1 cases were reported in patients younger than 20 years. Furthermore, only 14% of patients were older than 40 years, and there were no reports of patients older than 61. The H3N2 strain, on the other hand, was contracted across all age groupings. Approximately 27% of the reported H3N2 cases were in patients younger than 20 years, and 27% were reported in patients older than 40. More than 7% of the patients with symptomatic influenza caused by subtype H3N2 were older than 80 years. Both statistical measures confirmed a significant dissimilarity between the age distributions for the two subtypes: the probabilities that the observed age trends come from the same distribution are as low as P(MW) = 3.91E-05 and P(KS) = 4.81E-04 according to Mann-Whitney and Kolmogorov-Smirnov tests, respectively (Fig. 1).

**Figure 2:**
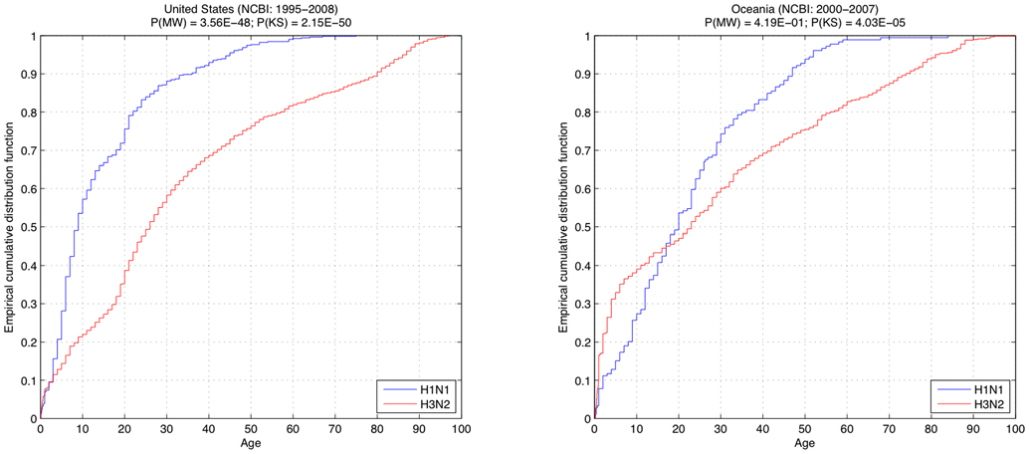
Empirical cumulative distribution of ages for patients with H1N1 (blue) and H3N2 (red), from the NCBI dataset, in the United States, between 1995 and 2008 (left) and Oceania, between 2000 and 2007 (right). Complementary to the results from New York State (Fig. 1), the low probabilities computed via Mann-Whitney (P(MN)) and Kolmogorov-Smirnov (P(KS)) tests show a significant difference between the distributions, which is spatially and temporally consistent.

When we examined the more extensive NCBI dataset for similar trends in age distribution among the subtypes, we observed the following in the 1995–2008 data from the United States (Fig. 2, left): approximately 76% of the H1N1 patients were younger than 20 years old, and less than 8% were older than 40. There was no report of any H1N1 patient older than 75 years in our dataset. On the other hand, roughly 39% of the H3N2 patients were younger than 20, and 32% were older than 40. Also, slightly less than 11% were older than 80; the oldest H3N2 patient was 97. Overall, ILI caused by H3N2 was exhibited across all age groupings, whereas H1N1 caused disease mainly in a younger population. The Mann-Whitney test applied to the NCBI dataset indicated a significantly low probability (P(MW) = 3.56E-48) that the age distribution was similar between H1N1 and H3N2. This was confirmed by the Kolmogorov-Smirnov test, with a very low probability of P(KS) = 2.15E-50.

Analysis of the Oceania dataset also demonstrated the contrasting age-wise distributions for H1N1 and H3N2. Although, due to a large number of H3N2 isolates from younger patients, and a higher variance in the data, the Mann-Whitney test failed to demonstrate the dissimilarities (P(MW) = 4.19E-1), the Kolmogorov-Smirnov test did show a significant difference (P(KS) = 4.03E-5) (Fig. 2, right).

**Figure 3:**
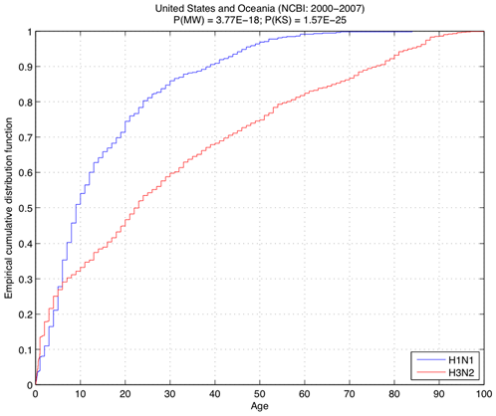
Empirical cumulative distribution of ages for patients with H1N1 (blue) and H3N2 (red), from the NCBI dataset in United States and Oceania combined, between 2000 and 2007. The low probabilities computed via Mann-Whitney (P(MN)) and Kolmogorov-Smirnov (P(KS)) further confirm the results shown in Fig. 2.

Furthermore, combining the Oceania and United States datasets from years 2000–2007, when there is available data from both geographical subsets, we see the same kind of disparate age trends between subtypes: P(MW) = 3.77E-18 and P(KS) = 1.57E-25 (Fig. 3).

**Figure 4:**
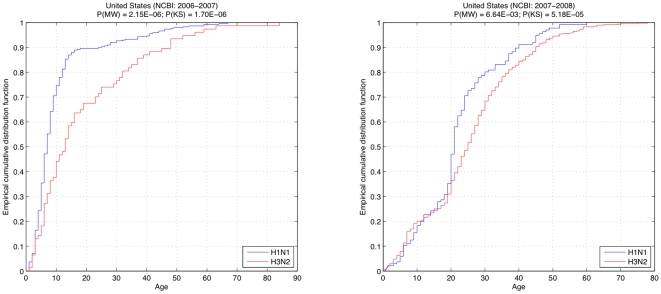
Empirical cumulative distribution of ages for patients with H1N1 (blue) and H3N2 (red), from the NCBI dataset, in United States during the influenza seasons of 2006–2007 (left) and 2007–2008 (right). The significantly low probabilities computed via Mann-Whitney (P(MN)) and Kolmogorov-Smirnov (P(KS)) tests during separate influenza seasons show the consistency in our results among sub-portions of the data and refute the possibility that the previous statistical results are due to a unique season.

To refute the possibility that the statistical results could have arisen due to a unique season, and to check the consistency of the results within subsets of the data, we also studied each season individually. The only two seasons in the United States for which the NCBI database provided data adequate for statistical analysis, were 2006–2007 and 2007–2008. We again observed different age trends between influenza subtypes in each of these seasons, and both the Mann-Whitney and Kolmogorov-Smirnov tests indicated low probabilities that the distributions were the same (Figs. 4).

**Figure 5:**
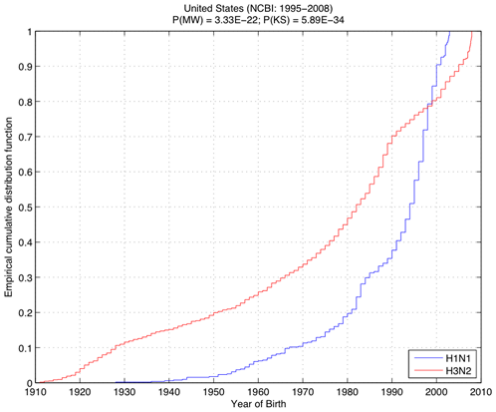
Empirical cumulative distribution of birth year for patients with H1N1 (blue) and H3N2 (red), from the NCBI dataset, in the United States, between 1995 and 2008. The significant statistical dissimilarity between the distribution for the year of birth (P(MW) = 2.04E-24 and P(KS) = 1.13E-35) hints to an existing immunity against one of the subtypes in different age groups, possibly carried over from a previous pandemic.

In addition, we evaluated the United States dataset from the NCBI based on the birth year of the patients, in order to investigate the correlation between the previous pandemics and possible immunity to one of the subtypes (Fig. 5). Four percent of the H1N1 patients were born before 1957 (the H2N2 pandemic), versus 24% of the H3N2 patients; 10% of the H1N1 patients were born before 1968 (the year of the H3N2 pandemic) versus 32% of the H3N2 patients. Also, 16% of the H1N1 patients were born before 1977 (the year of reemergence of H1N1), compared to 42% of the H3N2 patients. The significant statistical dissimilarity between the distributions of year of birth (P(MW) = 3.33E-22 and P(KS) = 5.89E-34) hints at an existing immunity against one of the subtypes in different age groups, possibly carried over from a previous pandemic.

## Discussion

We have analyzed the differences in patient age distribution between influenza A subtypes, using isolates from the 2006–2007 and 2007–2008 influenza seasons in New York State. These data show a compelling trend that younger persons exhibit ILI caused by the H1N1 subtype statistically more frequently than do older persons. We confirmed the results by combining large datasets for North America and Oceania, from 1995 to 2008, taken from the NCBI database. The same trend is observed for every year and for each geographical grouping. These results suggest that the two influenza subtypes, which are co-circulating around the globe, target two different age subpopulations with acute illness. These observations, made within geographically and temporally diverse datasets, are consistent with findings of the previous regional populations studies, conducted in the 1970's and 1980's, [7] that a lasting immunity to H1N1 subtypes of influenza virus, possibly carried over from exposure to previous epidemics and pandemics, exists in the older population that decreases their risk of developing the acute symptomatic disease.

These observations also complement some of the previous findings on the seasonal evolution of influenza A virus. For example, Rambaut et al. [16], who studied a population that was spatially and temporally similar to the one in our analysis, identified a weaker antigenic drift in H1N1, leading to a global co-circulation of multiple H1N1 lineages and weaker H1N1 bottleneck effects between seasons compared to those of H3N2. If H1N1 does preferentially target a younger population, as our results indicate, a lower antigenic pressure and less-severe bottlenecks in the viral population, are expected. Furthermore, the different host population of H3N2 could explain the subtype's lower diversity and more severe bottlenecks.

In addition, our analysis reflects the morbidity in the current population, which is also affected by the new H1N1 strain of influenza A virus that has been infecting humans since March 2009. Interestingly, most of the patients that show symptomatic disease from infection by this new strain are also young as 60% of the reported cases are 18 years old or younger [12], [17]. This is very similar to the distribution of age in seasonal H1N1 patients: 69% in the United States and 49% in Oceania (Figure 2) and 68% when the Oceania and United States datasets from years 2000–2007 were combined (Figure 3).

These results are especially pertinent for the assessment of risks in age-defined subpopulations. For example, in a year when H1N1 is predominant, public health resources should be focused on the younger populations, by introducing age-specific vaccines. From the point of view of epidemiological modeling, younger people have social patterns different from those of the older population, and it is likely that the two virus subtypes propagate differently in these distinct networks. This factor again has public health implications.
